# The Scientific American

**Published:** 1879-01

**Authors:** 


### THE SCIENTIFIC AMERICAN.

This journal as an embodient of the general science of the day, stands without an equal, or even a rival, in this country, and probably in the world.

To any one who desires to keep posted on the progress of general science it is indispensable. To inventors and those interested in inventions, it is *sine qui non.*

No one who is at all interested in either of these directions, can afford to be without the *Scientific American* and its *Supplement.* Every department of art and science comes within its scope.

In the department of mechanism, in this country, it has more influence in giving direction and stimulus than all other publications combined. The readers of the REGISTER can not better nor more profitably invest seven dollars than by subscribing for the *Scientific American* and the *Supplement.* The former alone is three dollars and twenty cents, and the latter, alone, five dollars, postage paid.

Send postal order or draft to the order of Munn & Co., 37 Park Row, New York.

